# The Relation of Noises to the Public Health1Read at the twenty-fourth annual meeting of the American Public Health Association, Buffalo, N. Y., September 18, 1896.

**Published:** 1896-10

**Authors:** William C. Krauss

**Affiliations:** Professor of nervous diseases, Medical Department of Niagara University, Buffalo, N. Y.


### THE RELATION OF NOISES TO THE PUBLIC HEALTH.[1]

By WILLIAM C. KRAUSS, M. D.,

Professor of nervous diseases, Medical Department of Niagara University, Buffalo, N. Y.

THE American people, as has been often asserted, are a patient and long-suffering nation, but when once thoroughly aroused to the magnitude of their grievances, are quick in meting out justice to their oppressors.

The residents of our cities have become accustomed to many inconveniences not experienced in rural communities, and have borne them patiently as an offset to the many advantages which they do enjoy. Such annoyances, or, better, nuisances, as the overcrowded and dark tenement, smoke, dirt and noise, have grown so amazingly in many cities during the past few years as to call for more than personal complaint to regulate them, and are attracting the attention of municipal authorities with a view of dealing with them from a judicial standpoint. Of all such nuisances that of noise seems to be the most difficult to abolish or mitigate, because of its omnipresence and close relationship with the affairs of every-

1. Read at the twenty-fourth annual meeting of the American Public Health Association, Buffalo, N. Y., September 18, 1896.

day life. Many such noises, however, could be easily suppressed and others decidedly moderated if proper attention were called to their uselessness as well as harmfulness, and if the authorities took the necessary steps looking toward their suppression. A certain amount of agitation and discussion is always necessary before such regulations are decided upon and enforced, and even before the community is aware of the fact that such noises are unnecessary and liable to regulation by municipal ordinances.

The only[1] references that the writer has been able to find on this subject are contained in editorials in the *Medical Record* of June 13, 1896, and in the *Maryland Medical Journal* of June 20, 1896, both taking their text from articles published in the New York daily papers of recent date.

It seems to me a propitious occasion, at this meeting of the American Public Health Association, to call attention to this growing nuisance and to ask this powerful body of scientific men to start the crusade against one of the most annoying and harassing drawbacks to comfort and good health in all our American cities.

That it is a question affecting the public health and, therefore, within the scope of this association to consider, the writer believes can be clearly shown, and although not a believer in the possibility of exterminating all noises, still a step in the direction named would decidedly moderate and, perhaps, abate some of the most annoying.

Sounds are impressions produced on the ear by vibrations of the surrounding elastic media, as air or water. Such sounds resolve themselves into two great classes : first, musical notes, and second, noises.

The musical sound is produced by sonorous shocks, taps or puffs, which follow each other at regular intervals with a sufficient rapidity of succession. A noise, on the other hand, is produced by an irregular and confused succession of sonorous shocks. While a musical sound flows smoothly without asperity or irregularity, the noise is jerky and produces jolting and jarring of the auditory nerve. A musical sound produces a feeling of pleasure and a regret when it ceases ; a noise, on the contrary, is attended with a sensation of irritation and a feeling of relief when it is terminated.

Noises are produced by a manifold variety of agents ranging

1. Since writing the above an article has appeared in the September number of the *North American Review*, by Dr. J. H. Girdner, on The Plague of City Noises ; also an editorial in the *Cincinnati Lancet-Clinic*, July 18, 1896.

all the way from the Chinese rattle to the thunderous discharge of atmospheric electricity.

To attempt a classification of the various noises and their provocative agents is a task not easily accomplished, and I trust you will bear with me in my rather hazardous undertaking. Noises may be divided into two great classes, those of nature or natural noises, and those of man or artificial noises.

- *Noise.*

  - Natural.

  - Artificial.

- *Natural.*

  - Thunder.

  - Storm.

  - Water.

  - Fire.

  - Catastrophe.

- *Artificial Noises.*

  - Necessary.

    - Warning.

    - Traffic.

    - Manufacture.

  - Unnecessary.

    - Summons.

    - Municipal.

  - Traditional.

    - Street Venders.

    - Celebrations.

    - Commotions.

    - Applause.

    - Small boy.

The natural noises stand in close relation with the elements, and may attain a degree of severity and terror surpassing all description. They emanate from sources over which man has but very little control and but slight power to check or to curtail them. They need, therefore, but passing notice and no consideration as regards their treatment. As such we have:

1. The deep, loud rumbling sounds heard after a flash of lightning, caused by the discharge of atmospheric electricity, and known as thunder.

2. The loud, roaring, often tempestuous sounds, due to more or less violent disturbances of the atmosphere, usually producing wind, rain, hail or snow storms. Sometimes they develop into cyclonic storms, passing from the warmer zones in a northeasterly direction and culminate at storm centers, producing such roaring and violent sounds as once heard will never be forgotten.

3. The somewhat musical and rhythmic sounds produced by the rushing, roaring and falling of waters. These sounds are more or less pleasing at the seashore and at waterfalls, but become terrifying and awe-inspiring in times of flood and tidal waves.

4. The crackling, snapping and explosive sounds attendant upon conflagrations.

5. Somewhat akin, though not properly included in this category, may be mentioned the various noises attendant upon catastrophies. As such may be mentioned earthquakes, volcanic eruptions, snowslides, floods, for which man is in no way responsible, and under the second head the collapse of buildings, bridges and other structures, collisions, caving in of mines and embankments, landslides and other accidental and distressing occurrences, for which man is in some measure responsible. These are usually attended with some loss of life and are, therefore, mingled with the groans and cries of the dying and wounded, making them the most dreaded and terrible of all the various noises.

The artificial noises may be divided into (1) the necessary, (2) the unnecessary, and (3) the traditional noises.

The necessary noises may be subdivided into (1) those of warning, (2) traffic, (3) manufacture.

*Warning.*—The noises of warning are manifested in various forms of bells, gongs, whistles, horns, sirens, and the like. They are a necessary evil to every street car, locomotive, fire apparatus, patrol wagon, ambulance, and their use is being extended to bicycles, trolley repair wagons, and in some cities to physician's carriages. The fog-horns and sirens on shore and on board are equally necessary, and until some other device is invented are indispensable to the safety and protection of many.

These noises all have for a common purpose the prevention of danger to mankind, either individually or collectively, and having a purpose should not be misused or disregarded, but carefully exercised and practised to the least extent commensurate with the public safety.

*Traffic.*—The noises of traffic are necessary only in so far as when they are reduced to a minimum. Their maximum development does not indicate the size or prosperity of the community, as some writers would like to infer, but a carelessness and perhaps indifference on the part of those either directly or indirectly concerned. The rumbling of cars, steam or electric, over poorly-built roadbeds, the rumbling of loosely-geared vehicles over roads and defective pavements, the puffing and prolonged whistling of locomotives, the rounding of curves by street cars are productive of extra noises which could be easily, if forcibly, moderated. In this city the noises arising from traffic over pavements is considerably reduced because of the wide extent of asphalt paving. Nevertheless we are behind some of our neighboring cities in the adoption of the rubber-tired carriage wheel, which in great measure reduces this form of noise to a minimum. These wheels are manufactured on a large scale in a nearby city, and their use should be encouraged by all who patronise this form of locomotion. The preference of the asphalted streets over the cobble-stone pavements has encouraged vegetation on the latter, and on many of our streets in the Elmwood district the grass is growing as luxuriantly as in some of the deserted villages of the far west. The incessant clatter of the gongs on street cars in the crowded business streets can be very easily avoided by the stationing of policemen at street crossings, as is done in this city, thus insuring safety to pedestrians, avoidance of collisions and the non-necessity of the gong. The avoidance of the business streets by the fire department, ambulances, bicycles and unnecessary vehicles of all description should be insisted upon as far as possible, and would relieve the congested business district of much noise, danger and confusion.

The value of real estate on some of our residence streets is seriously affected by the noise of the heavy trolley cars running over badly-repaired tracks and switches and around curves which are not kept properly oiled. The noise of a passing trolley car drowns conversation for the time being and makes veranda life in summer unpleasant and exasperating. Residents of the outlying districts try to secure homes near trolley lines, but try harder to get on streets not having the car service, their excuse being, partly if not wholly, to "avoid the noise, din and confusion of the discordant trolley." Much of this noise can be obliterated by keeping the tracks in good repair, the trucks and journals of the cars well oiled and tightened, and the speed limited in crossing streets, thus calling for less activity of the gong. All necessary noises will then be more cheerfully tolerated and the trolley line streets less discriminated against by home seekers.

The prolonged whistling of locomotives on approaching stations is unnecessary on roads having the block system, and the indiscriminate whistling and tooting of the locomotives in the yards and the steamers in the harbor might also be moderated, if not altogether silenced.

*Manufacture.*—The noises of the factories, mills, shops and forges, especially in the manufacture of metallic wares, are least liable to moderation, and must be patiently borne by those residing in their immediate neighborhood. Such factories should not be allowed upon closely settled residence streets, and the windows looking upon the streets should not be permitted to remain open. The plan adopted by Pittsburg, perhaps for commercial purposes merely, of having the rolling mills, forges, blast furnaces along the river banks or in the outlying suburbs, as at Homestead, Braddock and Duquesne, is an excellent one, and is more or less followed by the larger factories in this city.

*The Unnecessary Noises.— Summons.*—Such noises as the untimely ringing of church bells and the blowing of time whistles in the early morning hours, are no longer necessary in these days of universal time-pieces. In former years, when the time of day was watched by but few fortunate individuals, these noises were a necessity, but in recent years they have given rise to much complaint, and in this city a few have become silenced through the efforts of those who were annoyed and disturbed by them. The ringing of fire alarm bells is a source of much disturbance in many cities, and where a telegraph alarm system exists should be suppressed not only for the benefit of the citizens but firemen as well.

*Municipal Noises.*—The noisy, rattling iron trucks, used to convey garbage barrels from backyards to the street curb, constitute a source of much complaint, especially when such barrels are removed early in the morning; likewise the heavy and loosely geared street sweepers and dumping wagons as they move along the block pavements. Many other municipal noises could be mentioned which cause vexation and discomfort to those in the immediate neighborhood, especially in the early morning or evening hours.

*The Traditional Noises.*—By traditional noises I have designated those which are directly produced by individuals, many of which have become engrafted upon American city life. They are a nuisance if not a plague, and should certainly be curtailed if not altogether forbidden. Chiefly among these and enjoying, to a certain extent, police protection, are the cries of the street venders, as the newsboys, bootblacks, hucksters, "old clo. men," ragmen, the corner fakir, and, substituting a bell for the mouth, the scissor grinder, advertising wagons, and the like.

The "street arabs" of our large cities are a noisy and turbulent lot at the best, but when given license prove themselves a positive nuisance. The habit of crying out gruesome and sensational headings found in their papers by the newsboys should not for a moment be tolerated, and the congregating of these urchins about the street corners, all crying out in unison, should also be prohibited. In many European cities such noises are not allowed and it is doubtful whether the sale of the newspapers is injured by their suppression. Particularly provoking in this city is the noisy lot of newsboys set loose every Sunday afternoon with the New York Sunday papers, penetrating all parts of the city and crying out so lustily that they can be heard several blocks distant.

*Celebrations.*—The patriotism of the American youth is measured by his ability to make noise on the day of our national independence, and it seems on this day that youth still retains its grasp on the majority of our citizens. The newspapers throughout our land chronicle a long list of accidents on the day following—some the result of personal carelessness, some the outcome of runaways caused by the exploding firecrackers. Although patriotic and loyal to our country, still some other way might be found to celebrate this glorious holiday without the noise, explosions, fires and consequent accidents.

Political and other celebrations call forth much less demonstration, but the rule still holds good, "the more noise, the merrier."

*Commotions.*—The cries and noises of mobs, street brawls and night-hawks are recognised by our police authorities as unlawful, and offenders are punished sometimes for breaking the peace.

*Applause.*—The clapping of hands, stamping of feet, use of canes and umbrellas in public places, to express a feeling of gratification or sometimes one of impatience, is as universal, no doubt, as man himself. The writer recalls the disapproval which the stamping of feet always caused one of his professors at college and the expulsion from the class of all those implicated. It is, no doubt, a noisy and offensive custom and distasteful in other ways besides the noise which it creates.

Lastly, we meet the small boy with a thousand and one different ways of making a noise. His ability in this direction seems to be a criterion of his leadership among his comrades, and to rob him of these vested rights, even if we could, would make him less American and also less a boy. There seems to be no remedy but to avoid him, no recourse but his power of endurance, and he bids fair to remain lord of all he surveys.

Sounds which at times partake of the qualities of noise are those produced by domestic animals and birds. Especially annoying is it to be awakened at daybreak by the crowing of cocks, or during the midnight hours by the barking and crying of dogs or the concerted endeavors of several cats in the back yards. During the day pet parrots and other caged birds add their mite, sometimes their mightiest, to make life serious, and it is a question whether the crowded city is the proper domicile for many of these so-called pets.

---

We now turn to the second part of the paper and see what effect these varied noises, arising from widely different sources and of various degrees of pitch and intensity, have upon the public health.

The physiology of hearing is briefly as follows: The sound-waves are collected by the pavilion of the ear and conveyed to the tympanum by means of the external auditory canal. The tympanum vibrates in unison or in harmony with its fundamental tone, and these vibrations are carried along the ossicles to the labyrinth, where they are received by the organ of Corti. The impressions are conducted to the end filaments of the auditory nerve, thence along this nerve to the auditory center in the brain, located in the second temporal convolution. Here these impressions are collected, differentiated and interpreted and the conclusions dispatched to the various nerve centers for action. It is generally believed that the tympanum vibrates in resonance with sound-waves by influence, through the action of the small muscles in the middle ear. It is also undoubtedly thrown into vibrations by irregular waves of noise, and these latter demand more complex or irregular action than when the sounds are simple or melodious. The nerve filaments to these muscles acting upon the ossicles are extremely sensitive. Here, then, is one of the points of irritation produced by noises. The other is at the auditory center in the brain, where the complex impressions are to be interpreted. Single sounds call for little activity of the brain centers for their appreciation, but continuous complex noises demand a close application of these centers in differentiating the necessary from the unnecessary sounds, or in the interpretation of certain familiar or expected sounds among a lot of adventitious ones. The sensitiveness of the auditory center is well illustrated by the fact that hearing is the last of the special senses to be influenced by hypnotism, also in the administration of anesthetics; furthermore, that hearing is one of the first, if not the first, to recover after anesthesia, likewise in awakening from hypnotic sleep.

The dangers arising from noises are therefore two-fold. First, at the tympanum, where a certain amount of hyperemia and hypertrophy are liable to result, sometimes even rupture of the tympanum, producing partial or total deafness, and secondly, at the auditory center, where fatigue is apt to follow the incessant activity and irritation of these centers, and a general brain tire or brain exhaustion is liable to result. Whether or not noises are liable to produce inflammatory changes in the auditory nerve, the writer is unable to say.

The effects of noises upon the general system are varied, and in the short time remaining I will attempt only to enumerate the most important.

Noise is conceded to be a great disturber of personal comfort, and the hotel chosen as your headquarters was selected, as stated in the official journal,[1] because it is situated "in the quiet, residential portion of Buffalo, · · · and is free from *noise*, dust and smoke."

Comfort is that state of ease and rest so necessary to body or mind, and is preëminently one of the requisites of good health. Comfort and ease stand in the same relation as their antipathies, discomfort and disease, and if the absence of noise encourages the former, the presence of noise must stimulate the latter.

Noises have a tendency to unnerve an individual, especially when he is engaged upon some work calling for tact, coolness and good judgment. I know of no better illustration of this fact than the means usually resorted to by the rooters in our national game of base ball. At a critical moment in the game all the noise, cat-calls and groans that can be produced, are hurled at the pitcher, with the intention of rattling or unnerving him, and as a consequence he pitches wildly, batsmen are given bases on balls and the game is sometimes won thereby.

Strange noises sometimes produce a feeling of fear or inward excitement, accompanied with palpitation and feelings of distress. Whenever possible, professional men obliged to have their offices in the business district of the city, choose some side street free from traffic, or select quarters in the upper stories of our modern office buildings.

Perfect health demands a certain amount of quiet and repose not alone at night during sleeping hours, but during waking hours

1. The Municipality and County, special number, dedicated to the American Public Health Association, August, 1896, page 147.

as well. The active working brain is just as much in need of rest as are the muscles of the different portions of the body, and, perhaps, the most potent factor in accomplishing this need is to secure freedom from noise. The brain, as a whole, cannot be in perfect repose when certain centers are in a state of action or irritation. The association fibers, connecting all the various centers and parts of the brain, convey the irritation from one center to another and soon the whole brain is in sympathy with the disturbed and agitated center.

Sleep is one of the most important adjuncts to perfect health, and as has been so wisely said is "tired nature's sweet restorer." During slumber the restorative and constructive processes of the system are most actively engaged, and their interference by disturbed and broken sleep is sure to react detrimentally to the general system. The effects of rest and fatigue upon the ganglion cells of the brain and cord have been very carefully studied by C. F. Hodge, whose experiments are most interesting and instructive:

The ganglion cells, the active constituent of the nervous system, are situated in the gray cortex of the cerebrum and cerebellum, gray cornua of the myelon and in the spinal and peripheral ganglia. In the myelon they lie within the peri-ganglionic lymph spaces which vary in size according as the cells are in a state of rest or fatigue. When fatigued the protoplasm of the cells is shrunken and contracted and the lymph spaces are consequently larger and more conspicuous. As soon as the cells regain their normal rest and vigor they resume their usual size and the lymph spaces become correspondingly smaller. The nuclei of these cells behave in exactly the same manner, becoming larger or smaller according to the physical condition of the cells.[1]

In other words, "after prolonged rest the nerve cells are, so to speak, *charged;* they are full and ready for labor, but after a hard day's work, or after disturbed rest, they are *discharged*, shrunken and exhausted." (Gage.)

It is difficult to say just how many hours of sleep are necessary for each individual, but on the whole an average of eight hours should be the lowest limit. The various city noises are quite apt to cut this figure down perceptibly, especially in the early morning hours.

The long continued loss of rest and sleep is in the majority of cases followed by that *bête noir* nervousness or nervous prostration,

1. Krauss, Wm. C. The nerve elements in health and disease. Proc. Am. Mic. Soc., 1894.

or as physicians delight to call it, neurasthenia. Especially is this true of those individuals possessing an inherited neurotic tendency. I need not tell you what this all means, only to say that about the first advice given to the patient is to seek a quiet and peaceful spot either in the country, seashore or mountainside.

The effects of noises upon the organ of hearing itself have been referred to, and as an example the boilermaker's ear may be mentioned. Partial deafness is the rule among these mechanics, and their only hope is to secure other employment not attended with so much noise.

The effects of noises upon the sick are well known and hospitals are usually found away from the noisy districts of the city, or if the growth of the city has crept close to the hospitals the latter are protected by asphalt pavements, courts and by other means. Noises have a baneful influence over the sick, retarding convalescence, producing at times elevation of the temperature, exciting the nervous system and in some cases even bringing on convulsions. It is especially upon the nervous system that the consequences are most pronounced, and in such affections as tetanus, meningitis and encephalitis, the evil effects are most readily seen.

The covering of the pavement in front of houses containing sickness with tanbark is a common custom and only serves to prove some of the facts presented in this paper.

These, then, are some of the most important effects of noises upon the public health, and yet up to within a very short time ago no voice was raised, no pen was stirred in protest against their usurious tendencies. The American people, fast as they are, yet in matters of public health move too slowly and allow great odds to accumulate against them before they take the initiative.

The only remedy that I can suggest to abate many of these noises is to file protests and have your fellow-sufferers do likewise to the city health officer, and if he be the proper person in the proper place some measure of relief will be obtained.

---

Dribbling or Drooling of Infants.—Healthy infants never dribble or drool. This condition is due either to disorders of the digestive apparatus or to obstruction of nasal respiration, but is altogether unconnected with dentition.—De Silvera in *Lo Sperimentale.*

